# A practice-embedded, PDSA-guided implementation study of continuing professional development for stoma care in a resource-constrained setting

**DOI:** 10.3389/fmed.2026.1786649

**Published:** 2026-03-25

**Authors:** Ran Bi, Huixian Zhao, Lijun Tang, Rujie Zhou, Lianjie Liu

**Affiliations:** 1 Department of Oncology, Qinhuangdao First Hospital, Qinhuangdao, Hebei, China; 2 Qinhuangdao First Hospital, Qinhuangdao, Hebei, China; 3 Department of Gastroenterology, Qinhuangdao First Hospital, Qinhuangdao, Hebei, China

**Keywords:** continuing professional development, implementation study, nurse-led intervention, patient-reported outcomes, PDSA, practice-embedded quality improvement, resource-constrained settings, workplace learning

## Abstract

**Background:**

Continuing professional development (CPD) in resource-constrained clinical settings is frequently challenged by time pressures, staffing constraints, and limited integration between education and routine practice. Practice-embedded quality improvement approaches may offer a pragmatic pathway for aligning workforce development with care delivery. This study describes the implementation of a nurse-led, PDSA-guided practice improvement program designed to strengthen stoma care for patients with colorectal cancer.

**Methods:**

We conducted a single-site pragmatic implementation study using a quality improvement (QI) design. A Quality Stoma Practice Specific Program (QSPSP) was implemented in a tertiary medical center through three iterative Plan–Do–Study–Act (PDSA) cycles. The program incorporated specialist nursing leadership, standardized care processes, practice-based education activities, and interdisciplinary collaboration within routine workflows. Implementation and process indicators were assessed through structured audits and observational records. Patient-reported outcomes were explored using selected domains of the EORTC QLQ-C30 at post-discharge follow-up.

**Results:**

The QSPSP was integrated into routine nursing practice and was associated with improvements in care standardization, continuity of stoma care, infection control practices, and patient self-management education. Exploratory patient-reported outcome data from 27 participants indicated descriptive improvements in Emotional and Role Functioning domains at follow-up, while gains in Social Functioning were limited. Given the absence of baseline comparison and control conditions, these findings should be interpreted cautiously.

**Conclusion:**

This study provides a descriptive account of a practice-embedded, PDSA-guided implementation initiative in a resource-constrained setting. While professional development processes were embedded within the intervention, independent measurement of learning was not conducted. The findings highlight the feasibility of integrating structured practice improvement activities within routine clinical work and suggest potential implications for aligning CPD with implementation efforts in similar contexts. Further research with comparative designs and longer follow-up is required to evaluate clinical and educational outcomes more robustly.

## 1 Background

Continuing professional development (CPD) is widely regarded as an essential component of healthcare quality improvement, workforce adaptability, and patient safety. CPD is generally defined as a systematic, ongoing, and self-directed process through which healthcare professionals maintain and develop the knowledge, skills, attitudes, and competencies required for effective professional practice throughout their careers (1, 2). Distinct from episodic continuing education, CPD emphasizes long-term, practice-oriented learning, integrating formal educational activities with informal learning processes such as reflection, peer interaction, and experiential learning in the workplace. In this sense, CPD represents both an individual learning process and an organizational mechanism for professional capability development (3).

Beyond the maintenance of professional competence, CPD supports healthcare professionals in responding to changing clinical demands, adopting evidence-based practices, and sustaining improvements in care delivery. In nursing and other health professions, CPD has been associated with improved professional confidence, job satisfaction, and workforce stability, particularly in long-term care and resource-constrained settings where structured training opportunities are often limited (4). Increasingly, CPD is conceptualized not only as participation in accredited educational activities, but as a practice-embedded and career-oriented process aligned with clinical service delivery and organizational development.

Despite its recognized importance, CPD in many healthcare settings remains primarily delivered through discrete, classroom-based or workshop-style activities that are insufficiently integrated into routine clinical practice. Such approaches commonly presume the availability of protected learning time, adequate staffing, and institutional resources, which may not be feasible in resource-limited contexts. Consequently, gaps persist between professional learning and its application in everyday practice, limiting the impact of CPD on care quality and patient outcomes (5).

Recent literature in healthcare professions education and implementation science has emphasized practice-embedded, workplace-based CPD models as more feasible and contextually appropriate alternatives (6). Embedding CPD within routine clinical workflows may improve feasibility, acceptability, and sustainability, while strengthening the connection between professional learning, care processes, and patient-centered outcomes. However, empirical evidence evaluating such models—particularly studies incorporating implementation processes, professional practice changes, and patient-reported outcomes—remains relatively limited, especially in low-resource clinical environments.

In this context, the present study evaluated a nurse-led, practice-embedded CPD intervention implemented within a resource-constrained ostomy care pathway. Using a PDSA-guided approach, the study examined implementation processes, professional practice outcomes, and downstream patient-reported quality-of-life outcomes, with the aim of providing context-relevant evidence to inform the development of implementation-oriented CPD strategies in resource-limited healthcare settings.

## 2 Methods-design and evaluation of a practice-embedded CPD intervention

### 2.1 Study design, classification, and reporting framework

This study was designed as a pragmatic implementation study using a quality improvement (QI) approach, in which continuous professional development (CPD) functioned as a core implementation strategy to support practice change within routine nursing care. Specifically, the Quality Stoma Practice Specific Program (QSPSP) was conceptualized as a practice-embedded, workplace-based CPD intervention, operationalized through iterative Plan–Do–Study–Act (PDSA) cycles to facilitate learning transfer, professional competence development, and sustained implementation of evidence-based stoma care. PDSA model was selected due to its suitability for resource-constrained, nurse-led clinical environments requiring iterative refinement and rapid feedback. PDSA enables structured, small-scale testing of practice changes within routine workflows, supporting local adaptation while minimizing disruption to service delivery. Its widespread application in bedside quality improvement initiatives and integration with workplace-based CPD further supports its appropriateness for linking professional learning with real-time clinical practice transformation.

The evaluation adopted an implementation-oriented approach, focusing on feasibility, acceptability, fidelity, and practice-level change, alongside exploratory patient-reported outcomes. Consistent with the pragmatic aims of quality improvement research, the study did not seek to test hypotheses but to generate actionable learning regarding how CPD-informed implementation strategies can be integrated and adapted within resource-constrained clinical environments.

The reporting of this study was informed by the SQUIRE 2.0 guidelines for quality improvement studies and, where applicable, the Standards for Reporting Implementation Studies (StaRI), to enhance transparency, interpretability, and transferability of findings.

### 2.2 Implementation outcomes

In line with established implementation science literature, this study prioritized a focused set of implementation outcomes most relevant to the pragmatic aims and resource-constrained context of the intervention. Specifically, feasibility, adoption, and fidelity (process adherence) were selected as primary implementation outcomes, with acceptability assessed indirectly. Feasibility was prioritized to determine whether the practice-embedded CPD intervention could be delivered and sustained within routine clinical workflows under existing staffing and resource constraints. Adoption was emphasized to capture the extent to which core components of the intervention were taken up by nursing staff and integrated into everyday stoma care practices. Fidelity was assessed through structured audits and observational indicators to examine adherence to standardized care processes and CPD-related practice elements across PDSA cycles.

Acceptability was explored indirectly through sustained participation, engagement during PDSA cycles, and qualitative implementation reflections generated during routine team discussions, rather than through formal acceptability scales. Other implementation outcomes described in the literature (e.g., penetration, cost, sustainability) (7, 8) were not prioritized due to the formative, pilot nature of the study and the limited timeframe of the intervention.

### 2.3 Practice setting and resource context, and participants

The study was conducted at a single tertiary medical center in Hebei Province, China, providing surgical care for patients with colorectal cancer (CRC). In accordance with national expert consensus, protective stoma creation is commonly employed to reduce the clinical consequences of anastomotic leakage in CRC surgery. Despite this, routine stoma care within the institution was characterized by variability in nursing competencies, inconsistent education practices, and fragmented interdisciplinary coordination, reflecting constraints commonly encountered in resource-limited clinical settings.

The QSPSP was implemented within the colorectal surgery wards and involved 21 registered nurses, including 7 certified stoma nurses and 14 general ward nurses, who participated in practice-based educational and implementation activities to varying degrees depending on clinical role and shift allocation, as part of routine care. Nurses represented a range of professional experience levels (median years of practice: 7; range: 3–20).

Patient-reported outcome data were collected from 27 adult patients who underwent stoma creation during the active implementation phases of the PDSA cycles and met inclusion criteria for participation in quality-of-life assessment. Family caregivers were indirectly involved through structured education sessions but were not formal study participants.

The overall study duration was 27 months, encircling program development, three iterative PDSA cycles, and a consolidation phase. Each PDSA cycle spanned approximately 7–9 weeks, allowing sufficient time for implementation, observation, feedback, and iterative refinement.

### 2.4 CPD intervention and implementation strategy: quality stoma practice specific program (QSPSP)

The Quality Stoma Practice Specific Program (QSPSP) was developed as a practice-embedded CPD intervention integrated into routine nursing workflows. The program addressed empirically identified gaps in stoma care delivery, including communication of patient rights, access to specialist stoma nursing expertise, availability of standardized infection control resources, interdisciplinary coordination, and structured education for patients and family caregivers.

**Figure 1:**
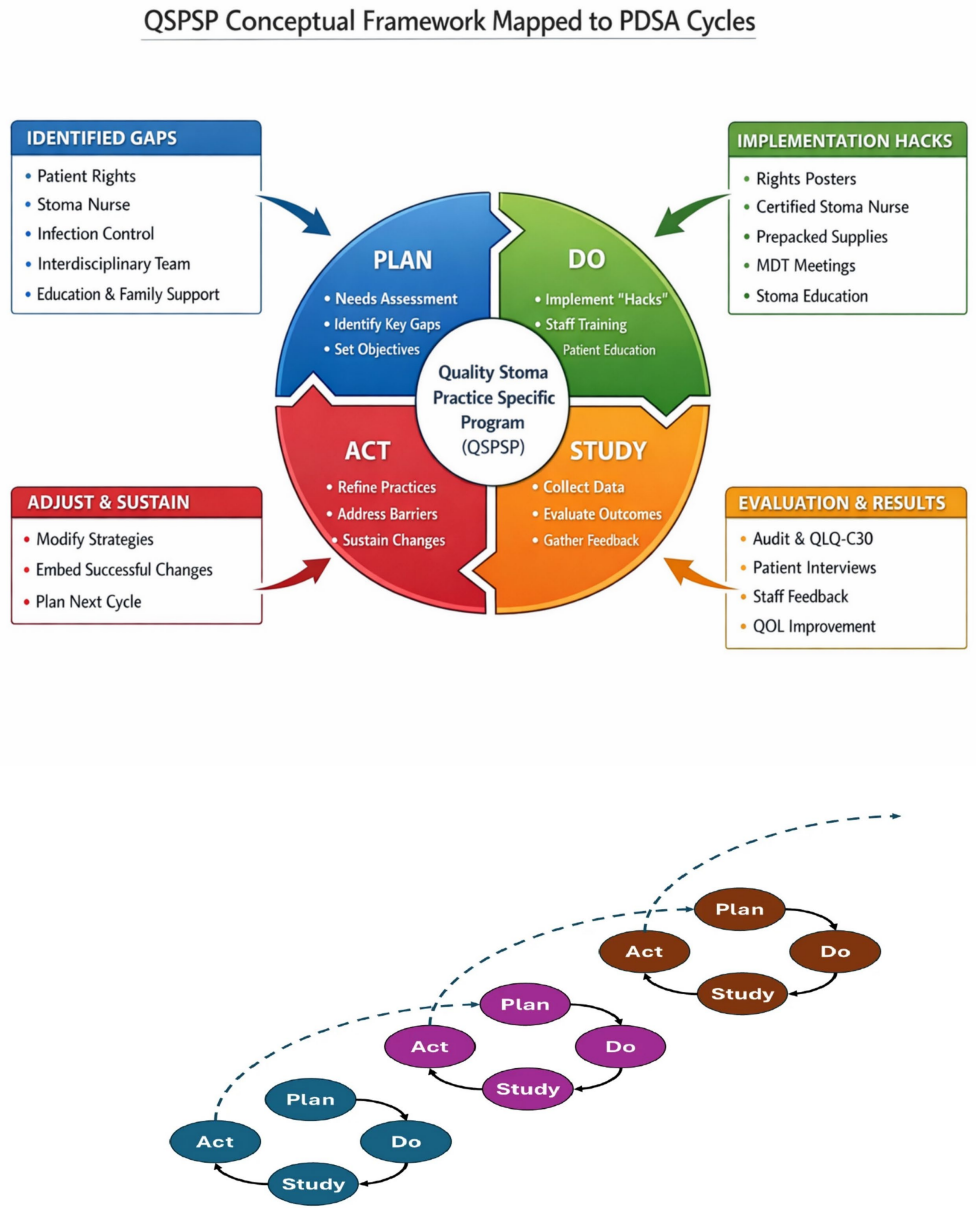
Conceptual and operational structure of the QSPSP. The upper diagram illustrates the conceptual framework of the QSPSP as a practice-embedded continuing professional development (CPD) intervention, highlighting its core components, including specialist nursing leadership, standardized care processes, workplace-based learning, and interdisciplinary collaboration. The lower diagram indicates the operationalization of this framework through three iterative Plan–Do–Study–Act (PDSA) cycles. These cycles represent the implementation mechanism through which conceptual elements were translated into practice, refined through audit and feedback, and progressively embedded into routine clinical workflows. The two layers are intentionally presented as conceptually distinct but functionally integrated: the upper layer reflects the theoretical and structural design of the intervention, while the lower layer illustrates its iterative enactment and adaptation within the clinical setting. The framework components were mapped onto successive PDSA cycles rather than aligned in a one-to-one structural correspondence.

Implementation occurred through three iterative PDSA cycles, enabling continuous adaptation in response to real-time feedback. Figure 1 illustrates the alignment of QSPSP components with the PDSA framework. The Plan phase involved prioritizing care gaps and designing targeted strategies; the Do phase focused on implementation within routine practice; the Study phase involved structured review of process and outcome indicators; and the Act phase emphasized refinement, contextual adaptation, and consolidation into routine workflows.

Although each cycle incorporated all four PDSA elements, planning predominated in Cycle 1, implementation and testing in Cycles 1–2, and evaluation and consolidation in Cycle 3. In the final cycle, the Act phase focused on institutionalization rather than initiation of a subsequent cycle (Table 1).

**Table 1:** Phases of the QSPSP implementation process guided by PDSA cycles.

Dominant PDSA phase by cycle	Key activities	Primary stakeholders	Outputs/deliverables	Lessons learned
Cycle 1—PLAN	Needs assessment; baseline audit; patient and caregiver interviews; QLQ-C30 baseline assessment	Nurse managers, surgical nurses, patients, caregivers	Identified priority gaps; defined QSPSP objectives and action areas	Early stakeholder engagement clarified feasible, context-specific targets
Cycle 1–2—DO	Introduction of stoma nurse role; implementation of education materials; establishment of MDT huddles; deployment of infection control kits	Certified stoma nurse, surgeons, MDT members	Standardized stoma care pathway; structured education processes	Small, low-cost “hacks” enabled rapid uptake and staff buy-in
Cycle 2–3—STUDY	Process audits; staff reflective feedback; patient and family interviews; follow-up QLQ-C30 assessment	Nursing staff, patients, quality team	Evidence of improved care consistency and selected QOL domains	Process indicators improved faster than patient-reported outcomes
Cycle 3—ACT	Refinement of underperforming strategies; integration into routine practice; planning next PDSA cycle	Nurse leadership, MDT	Sustained successful practices; revised protocols	Sustainability requires role clarity and leadership support

### 2.5 Practice-embedded CPD and workplace learning processes

Educational activities were delivered through workplace-based learning mechanisms, including bedside mentoring, targeted skills reinforcement, standardized educational materials, and structured interdisciplinary discussions. Rather than functioning as discrete training events, CPD activities were embedded within daily clinical routines, enabling experiential learning, reflective practice, and immediate application to patient care.

**Figure 2:**
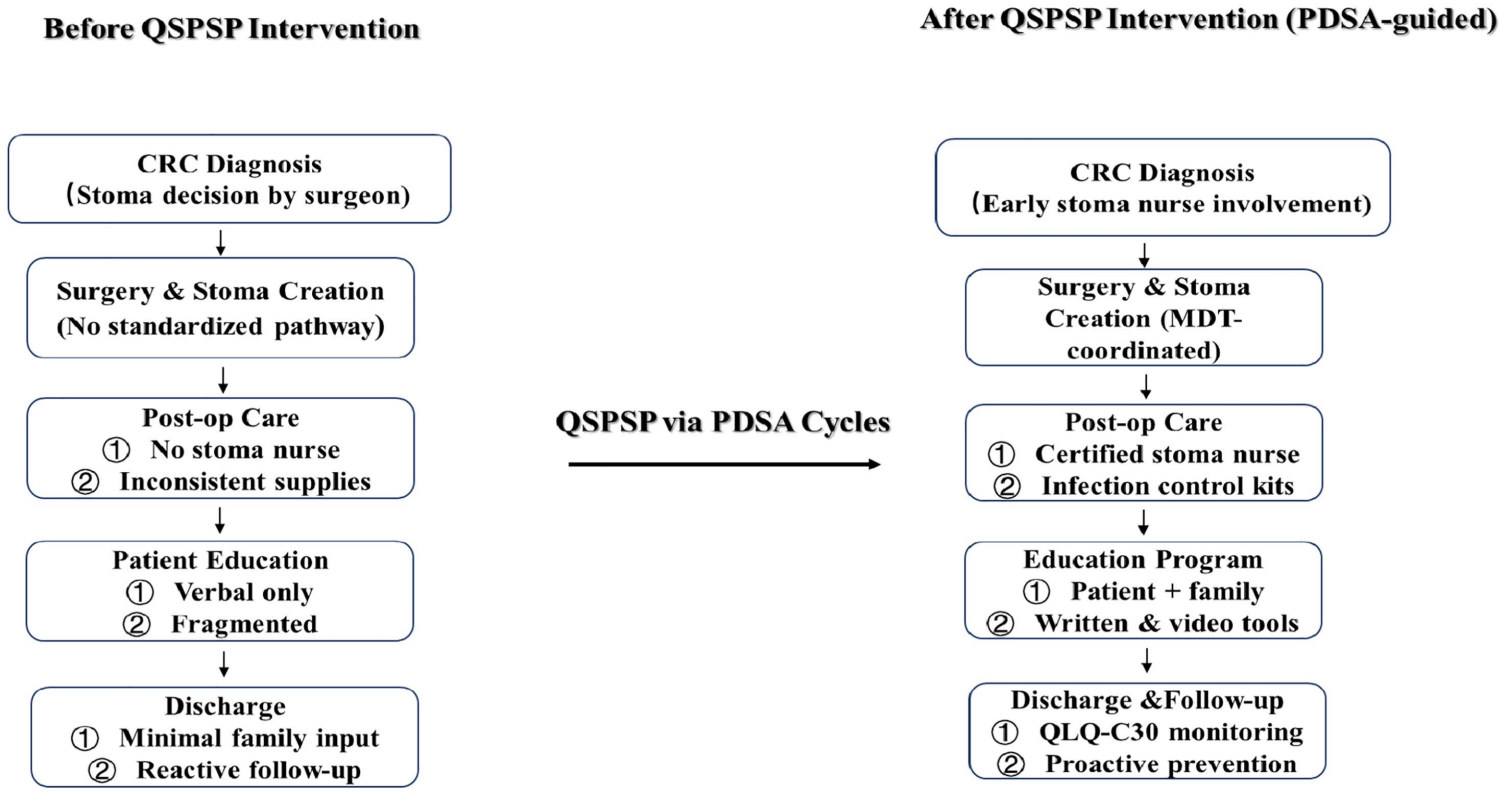
Stoma care pathway before and after implementation of the Quality Stoma Practice Specific Program (QSPSP) guided by PDSA cycles.

This approach was explicitly designed to support learning transfer, allowing nurses to integrate new knowledge and skills into practice in real time. Figure 2 shows the transition from a surgeon-led, fragmented care model to a certified stoma nurse–led, interdisciplinary pathway with standardized education, proactive complication prevention, and continuous monitoring of patient quality of life.

### 2.6 Implementation, professional learning, CPD-related, and patient outcome measures

Implementation and practice-change outcomes were assessed using structured audits, observational checklists, and predefined process indicators, including adherence to standardized care procedures, documentation completeness, interdisciplinary communication, and delivery of patient education.

Implementation and process indicators were operationalized as predefined audit items reflecting the presence, consistency, or completeness of key practice components (e.g., documentation elements, standardized education delivery, infection control practices, interdisciplinary coordination). For each indicator, percentage values represent the proportion of observed cases, audited records, or care episodes meeting predefined criteria during a given PDSA cycle or at post-implementation review. These percentages were derived from routine practice audits and structured observational checklists completed by the project team members and reviewed collaboratively during PDSA “Study” phases. The indicators were used to support formative assessment, practice-based learning, and iterative refinement of the intervention rather than to generate comparative or inferential statistics.

In this practice-embedded CPD model, professional learning was not assessed through formal examinations or psychometric instruments. Instead, learning was inferred from observable practice change and professional role development within the clinical setting. Indicators included increased adherence to standardized stoma care protocols, assumption of specialist-led mentoring responsibilities, improved documentation consistency, and active participation in structured reflective discussions during PDSA review meetings. These performance-based indicators were interpreted as evidence of learning transfer and professional competence development embedded in routine practice. Formal educational assessments were not conducted, consistent with the pragmatic and workplace-based nature of the intervention.

Patient-reported outcomes were explored using selected domains of the EORTC QLQ-C30, including Emotional Functioning, Role Functioning, Social Functioning, and Global Health Status. These domains were selected *a priori* based on their conceptual relevance to stoma care, which commonly involves psychological adjustment, restoration of daily functional roles, social reintegration, and overall perceived health status following colorectal surgery. The QLQ-C30 was administered at discharge and during the routine post-discharge outpatient follow-up period to align with standard clinical evaluation time points and to capture early adaptation experiences while minimizing additional patient burden. The instrument was used as an exploratory indicator of downstream patient experience with care quality and continuity rather than as a definitive clinical effectiveness endpoint. Patient-reported outcome data were obtained from 27 adult patients who completed the intervention pathway. The QLQ-C30 was administered at follow-up within the post-discharge period (typically during scheduled outpatient review or structured telephone follow-up approximately 2–4 weeks after discharge), allowing preliminary assessment of early adaptation and care continuity following stoma surgery.

Table 2 summarizes identified care gaps and the corresponding pragmatic implementation strategies (“implementation hacks”) developed and refined across PDSA cycles.

**Table 2:** Identified gaps and corresponding implementation hacks within the QSPSP (PDSA-guided).

Identified gap	Root cause (qualitative insight)	Implementation “hack”	PDSA phase(s) applied	Intended outcome
Limited awareness of patient rights	Rights not displayed; explanations inconsistent	Visual patient-rights posters; bedside rights checklist during admission	Plan, Do	Improved patient awareness and perceived respect
Absence of certified stoma-specific nurse	No designated role; reliance on general surgical nurses or caregiver	Appointment and training of a lead certified stoma nurse; protected clinic time	Plan, Do, Act	Continuity of care; improved technical confidence
Inconsistent infection control and stoma supplies	Fragmented procurement; lack of standard kits	Pre-packed stoma care kits; ward-level stock monitoring	Do, Study	Reduced infection risk; standardized care
Poor interdisciplinary coordination	Siloed workflows; lack of routine communication	Weekly MDT meetings; standardized referral triggers	Do, Act	Integrated, patient-centered care
Fragmented patient education	Verbal-only education; time constraints	Illustrated booklets; stepwise self-care education; video resources	Do, Study	Enhanced patient self-management
Limited family/caregiver engagement	No structured family education; low health literacy	Dedicated family education sessions; plain-language materials	Do, Study	Improved caregiver preparedness
Suboptimal post-operative QOL domains	Focus on clinical recovery only	Routine QLQ-C30 assessment to guide care adjustments	Study, Act	Improved emotional and role functioning
Inconsistent nursing care documentation	Heavy workload; unclear expectations	Simplified stoma care documentation templates	Act	Improved care continuity and auditability

### 2.7 Data sources, analytic approach, and use of qualitative inputs

Process and audit data were reviewed descriptively and comparatively at the conclusion of each PDSA cycle to inform iterative refinement of the QSPSP. Patient-reported outcome data were summarized descriptively to explore trends over time.

Qualitative inputs—including informal staff feedback, reflective discussions during PDSA reviews, and observational field notes—were used as implementation reflections to contextualize quantitative findings and identify barriers and facilitators to practice change. These data were not subjected to formal qualitative coding but were systematically reviewed by the implementation team to inform adaptive decision-making, consistent with the pragmatic aims of quality improvement research.

In addition to quantitative and process indicators, qualitative observations arising from audits, team reflections, and informal feedback during PDSA “Study” phases were documented to inform iterative refinement of the intervention. These qualitative inputs were used as implementation reflections to contextualize observed changes and identify practical barriers and facilitators, rather than being subjected to formal qualitative data collection or analytic procedures.

### 2.8 Ethical considerations

This study was reviewed and approved by the Ethics Review Committee of the participating medical center in Hebei Province, China (Approval No. C20240004). All procedures were conducted in accordance with the Declaration of Helsinki and relevant national regulations governing research involving human participants.

As a practice-based quality improvement initiative, the intervention was implemented within the scope of routine clinical care. Institutional approval was obtained prior to program initiation. Nursing staff were informed of the study objectives, procedures, and CPD-oriented nature of the project, and participation in educational and practice-related activities was voluntary.

Patients whose care data and patient-reported outcomes were included received clear verbal and written information regarding the study purpose, data collection procedures, and use of anonymized data for research and publication. Written informed consent was obtained prior to completion of patient-reported outcome measures. Participants were informed of their right to decline participation or withdraw at any time without impact on care.

All data were de-identified prior to analysis to ensure confidentiality. Access to study data was restricted to the research team, and data were stored securely in accordance with institutional data protection policies.

## 3 Results

### 3.1 Implementation and practice change outcomes

Unless otherwise specified, percentage values reported below reflect the proportion of audited care episodes or documentation records meeting predefined implementation or practice criteria at each stage of the PDSA-guided intervention.

Across three iterative PDSA cycles, implementation of the Quality Stoma Practice Specific Program (QSPSP) was associated with measurable improvements in nursing practice, care processes, and practice-based learning integration. Enhanced visibility and structured communication of patient rights were consistently observed following implementation, contributing to increased patient awareness and perceptions of respect during hospitalization. The introduction of a certified stoma-specific nurse functioned as a key practice facilitator, strengthening continuity of care and serving as an in-situ educational resource for nursing staff. Patients frequently attributed increased confidence and perceived competence in stoma management to consistent access to specialist nursing expertise.

Standardization of infection prevention practices—most notably through the introduction of pre-packaged stoma care kits—improved consistency in care delivery and supported early identification, prevention, and management of peristomal skin complications, particularly excoriation-related dermatitis. These changes reflected improved alignment between evidence-based protocols and routine clinical practice. Structured patient self-care education, supported by standardized written and visual materials, enhanced patients’ perceived readiness to perform stoma care after discharge and reinforced nurses’ educational roles within everyday workflows.

Interdisciplinary collaboration improved following the introduction of routine multidisciplinary discussions embedded within clinical care processes. However, engagement remained variable, reflecting ongoing operational constraints and competing clinical priorities. Family caregiver involvement increased through targeted education sessions, although variability in comprehension persisted, particularly among caregivers with lower health literacy. Despite the introduction of simplified documentation tools intended to support learning transfer and practice standardization, completion of comprehensive nursing care plans remained inconsistent. Documentation gaps were most evident in the systematic recording of common stoma-related complications, including retraction, mucocutaneous separation, parastomal hernia, prolapse, bowel obstruction, bleeding, constipation, diarrhea, and peristomal skin breakdown. Improvements in interdisciplinary collaboration were assessed through structured observational records and participation logs maintained during PDSA cycles. Indicators included frequency of joint case discussions, documented cross-disciplinary consultations, and participation of surgeons and nursing staff in audit-review meetings. These measures were used as process indicators reflecting coordination and communication rather than formal social network or team-climate metrics.

In addition to implementation indicators, several observed changes were specifically attributable to CPD mechanisms embedded within the intervention. Standardization of stoma care procedures and improved documentation completeness were closely linked to structured mentoring by the specialist nurse, case-based bedside teaching, and reflective discussions conducted during PDSA review meetings. Increased consistency in patient education delivery and the emergence of nurse-led teaching behaviors (e.g., peer instruction, role modeling, and protocol clarification during routine care) further reflected practice-based professional development rather than solely procedural compliance. These patterns suggest that CPD mechanisms—particularly mentoring, situated learning, and iterative feedback—functioned as active drivers of professional role development and sustained practice change.

### 3.2 Patient-reported quality of life outcomes (EORTC QLQ-C30)

Patient-reported quality of life outcomes were examined to assess whether practice-level changes associated with the QSPSP translated into perceived patient benefit. Following program implementation, improvements were observed in the Emotional Functioning and Role Functioning domains of the EORTC QLQ-C30. Patients reported reduced anxiety related to stoma care and greater capacity to engage in daily activities, suggesting that enhanced nursing support and education contributed to improved psychological adaptation and functional recovery.

In contrast, changes in Social Functioning were limited over the study period, indicating persistent challenges related to social participation and longer-term adjustment following stoma creation. Improvements in Global Health Status/Quality of Life were modest and varied across individuals, suggesting that broader quality-of-life adaptation may require sustained, longitudinal support extending beyond the initial implementation and inpatient phases of care.

### 3.3 Summary of achieved and unmet targets

Overall, the QSPSP was successfully embedded into routine clinical practice, with several predefined implementation and practice targets achieved and others partially met or unmet. Achieved outcomes reflected improved alignment between evidence-based stoma care standards, nursing practice, and workplace-based learning. Areas not fully meeting targets—particularly interdisciplinary engagement, documentation consistency, and social functioning outcomes—were explicitly reviewed during the final ACT phase. These findings informed refinement strategies and identified priority areas for subsequent PDSA cycles, reinforcing the iterative, learning-oriented nature of the CPD-informed implementation approach. A summary of achieved outcomes and unmet targets is presented in Table 3. Achievement status classifications were determined based on predefined audit thresholds established at project initiation. Specifically, status levels (e.g., averaged adherence rates >60% were classified as “*Achieved*,” 40–60% as “*Partially Achieved*,” and <40% as “*Unmet*”) reflected the proportion of audited cases meeting standardized criteria during post-implementation review. These categorizations were applied during PDSA “Study” phases to guide iterative refinement and represent formative implementation assessments rather than inferential statistical comparisons. Unless otherwise specified, percentage values reported in Table 3 and in this section reflect the proportion of audited care episodes or documentation records meeting predefined implementation or practice criteria at each stage of the PDSA-guided intervention.

**Table 3:** Outcomes achieved versus unmet targets following implementation of the QSPSP.

Domain	Intended target	Outcome achieved	Status (range)^*^	Explanatory notes (qualitative insight)
Patient rights awareness	Patients understand and can articulate their rights	Increased visibility and reported awareness	Achieved (47–80%)	Posters and bedside explanations were consistently cited as helpful
Stoma-specific nursing care	Continuous, specialized stoma support	Certified stoma nurse integrated into care pathway	Achieved (34–87%)	Patients valued continuity and technical expertise
Infection control practices	Standardized infection prevention during stoma care	Improved availability and adherence	Achieved (61–84%)	Pre-packed kits reduced variability in practice
Patient self-care education	Patients demonstrate confidence in stoma self-management	Improved self-care competence	Achieved (67–90%)	Written and visual materials enhanced understanding
Interdisciplinary collaboration	Regular MDT involvement in stoma care	Improved coordination but inconsistent attendance	Partially achieved (34–51%)	Scheduling conflicts limited full participation
Family/caregiver engagement	Relatives understand care needs and support patients	Improved but variable understanding	Partially achieved (51–69%)	Health literacy and time constraints were barriers
Nursing care plan documentation	Complete and consistent documentation	Inconsistent completion	Not fully achieved (27–44%)	Competing workload priorities affected compliance
Quality of life (QLQ-C30)—emotional functioning	Improvement in emotional well-being	Moderate improvement observed	Achieved (64–84%)	Patients reported reduced anxiety over time
Quality of life (QLQ-C30)—role and social functioning	Return to usual roles and social activities	Limited improvement	Not fully achieved (27–44%)	Adaptation to stoma required longer-term support
Sustainability of practice changes	Integration into routine clinical care	Core elements sustained; some require reinforcement	Partially achieved (41–63%)	Ongoing leadership support identified as essential

^*^Percentages indicate the proportion of audited care episodes or documentation records that met predefined implementation or practice criteria at the corresponding assessment point; values are reported for formative evaluation and are not intended for inferential comparison. Ranges indicate the lowest and highest proportions of audited care episodes or documentation records meeting predefined criteria across successive PDSA cycles during the implementation period.

## 4 Discussion

This study examined the implementation of a Quality Stoma Practice Specific Program (QSPSP), guided by the Plan–Do–Study–Act (PDSA) model, as a practice-embedded continuing professional development (CPD) and implementation strategy to improve stoma care for patients with colorectal cancer. The findings indicate that a structured, iterative, workplace-based approach can facilitate the translation of evidence into routine nursing practice, resulting in observable changes in care processes and selected patient-reported outcomes. These results align with prior evidence demonstrating that structured, continuous care and education programs improve stoma-related self-efficacy, quality of life, and patient satisfaction while reducing complications compared with routine care alone (9).

Importantly, this study extends existing literature by illustrating how an established quality improvement methodology can be conceptualized and operationalized as a CPD intervention, supporting learning transfer and professional competence development within complex clinical environments. Rather than functioning solely as a process improvement tool, the PDSA framework served as an organizing structure for iterative learning, reflection, and adaptation embedded in everyday nursing practice.

### 4.1 Educational and CPD outcomes: learning transfer and professional role development

The QSPSP indicated meaningful educational and CPD-related outcomes by facilitating learning transfer within routine clinical practice. Through iterative PDSA cycles, nurses engaged in repeated processes of planning, enactment, observation, and reflection, allowing newly acquired knowledge and skills to be immediately applied, tested, and refined in real-world care contexts. This practice-embedded learning structure aligns with contemporary CPD scholarship, which emphasizes experiential, workplace-based learning as a more effective mechanism for sustaining professional behavior change than standalone, didactic educational approaches.

A key educational outcome of the intervention was the strengthening of professional competence and role confidence, particularly among certified stoma nurses. Within the QSPSP, specialist nurses functioned not only as technical experts but also as facilitators of learning transfer, providing bedside mentorship, reinforcing standards of care, and supporting reflective practice during routine clinical work. This dual clinical–educational role enabled tacit knowledge to be shared and contextualized, supporting progressive skill development among general ward nurses. Such specialist-led facilitation is increasingly recognized as a critical enabler of effective CPD, especially in resource-constrained settings where formal training opportunities may be limited, and is consistent with international recommendations identifying specialist nursing expertise as foundational to high-quality ostomy care (10).

Interdisciplinary engagement within the QSPSP also contributed to CPD outcomes by exposing nurses to shared decision-making processes and collaborative problem-solving. While structured multidisciplinary discussions enhanced awareness of team-based care principles, variability in participation highlighted persistent organizational barriers to sustained interprofessional learning. From a CPD and implementation perspective, this finding reflects common challenges in translating collaborative learning intentions into routine practice, particularly in environments characterized by competing clinical demands and limited protected time. Similar constraints have been reported in prior studies of interdisciplinary CPD initiatives, underscoring the importance of supportive organizational structures for enabling learning across professional boundaries (11).

Finally, the integration of patient- and family-centered education within the QSPSP reinforced nurses’ educational competencies and pedagogical confidence. Delivering structured yet flexible education at the bedside required nurses to adapt communication strategies to individual patient and caregiver needs, thereby strengthening applied teaching skills as part of everyday practice. Consistent with previous research, such contextualized educational engagement supports professional development in psychosocial care and patient empowerment, even when broader quality-of-life outcomes may require longer-term follow-up to fully manifest (11, 12).

Collectively, these findings indicate that the QSPSP functioned not only as a clinical improvement initiative but also as a structured CPD intervention that supported experiential learning, professional role development, and the transfer of evidence-based knowledge into sustained nursing practice.

### 4.2 Clinical and process outcomes: practice standardization and care delivery change

Beyond educational effects, the QSPSP yielded observable improvements in clinical processes and care delivery at the practice level. Standardization of infection control procedures, increased availability of pre-packaged stoma care kits, and clearer role delineation within nursing workflows contributed to greater consistency in routine stoma care. These changes reflect successful translation of evidence-based recommendations into everyday practice through structured, PDSA-guided implementation.

Improvements in care processes were particularly evident in domains that were closely aligned with existing workflows and could be readily operationalized, such as infection prevention practices and patient education delivery. In contrast, documentation completeness and sustained interdisciplinary participation remained variably achieved. From an implementation perspective, this pattern is consistent with prior findings indicating that practice changes requiring additional cognitive or administrative effort—such as comprehensive documentation or coordination across professional boundaries—are more sensitive to workload pressures and competing priorities.

Importantly, partial or unmet clinical targets should not be interpreted solely as shortcomings but rather as indicators of contextual constraints within real-world practice environments. Within the PDSA framework, such implementation gaps functioned as actionable learning signals, guiding iterative refinement of tools, expectations, and workflows. Consistent with implementation science literature, these findings underscore the importance of aligning practice change initiatives with organizational capacity, leadership support, and existing clinical routines to support sustained process improvement (13).

### 4.3 Patient-reported outcomes: downstream signals of care experience

Patient-reported outcomes provided descriptive insight into early postoperative experience following implementation of the QSPSP. Improvements observed in the Emotional Functioning and Role Functioning domains of the EORTC QLQ-C30 were temporally associated with the implementation period and may reflect enhanced continuity of care, structured education delivery, and increased nursing competence within the clinical workflow. However, given the study design, no causal inference regarding intervention effectiveness can be established.

The limited change observed in Social Functioning and Global Health Status further suggests that these dimensions of quality of life are influenced by broader psychosocial, environmental, and temporal factors that extend beyond the scope of hospital-based implementation activities (12). Adaptation to stoma creation is a complex and longitudinal process, and meaningful social reintegration may require sustained community and family support beyond the early postoperative window.

### 4.4 Integrating educational, clinical, and patient signals: an implementation perspective

Taken together, the findings provide a descriptive account of how a structured, PDSA-guided quality improvement initiative was embedded within routine nursing practice in a resource-constrained setting. Educational activities, specialist nurse leadership, audit–feedback processes, and standardized workflows were integrated into daily care delivery, contributing to observable changes in clinical processes and documentation practices.

While professional development processes were intentionally incorporated into the intervention design, independent measurement of learning outcomes was not undertaken. Accordingly, improvements in adherence to care standards and participation in structured reflection should be interpreted as indicators of practice change rather than direct evidence of educational effect. The study therefore illustrates how CPD-oriented elements can be operationalized within implementation activities, but does not establish causal relationships between learning and observed process improvements.

Patient-reported outcomes offered exploratory insight into early post-discharge experience. Descriptive improvements were observed in Emotional and Role Functioning domains at follow-up; however, in the absence of baseline measurement, comparator groups, or longitudinal tracking, these findings cannot be attributed to the intervention and should be interpreted cautiously (14, 15). Broader domains such as Social Functioning appeared less responsive within the study timeframe, highlighting the complex and socially mediated nature of longer-term adaptation following stoma creation.

### 4.5 Conceptual positioning within an implementation perspective

Although formal implementation science frameworks were not prospectively applied in the study design, selected constructs from established frameworks such as CFIR and ERIC provide a useful retrospective interpretive lens. Within the inner setting, workload pressures, documentation burden, and limited protected time influenced the pace and variability of practice change, while leadership endorsement and specialist nurse presence functioned as enabling conditions (16). These observations are consistent with prior implementation research emphasizing the importance of organizational readiness and facilitative leadership (17).

At the level of process, the iterative structure of PDSA cycles created recurring opportunities for planning, reflection, and adaptation within routine clinical workflows. Rather than serving solely as a quality improvement tool, PDSA functioned operationally as a structured mechanism for embedding reflective practice within care delivery. However, because learning outcomes were not independently measured, the educational implications of this structure remain inferential and warrant further empirical investigation.

To clarify transferability, it is helpful to distinguish adaptable components from structural features likely necessary for replication. Specialist nurse leadership, integration of educational activities within routine work, iterative audit–feedback cycles, and baseline organizational endorsement appear to represent foundational elements. Other operational details, including documentation formats and workflow sequencing, may reasonably vary across contexts.

Overall, this study contributes a descriptive implementation account demonstrating the feasibility of integrating structured practice improvement activities within routine nursing work in a resource-constrained hospital setting. Future research employing prospective framework-guided design, independent educational assessment, and comparative or longitudinal methodologies is needed to more rigorously evaluate clinical and professional development outcomes.

## 5 Limitations

This study has several limitations that warrant careful consideration when interpreting the findings. First, the intervention was conducted within a single tertiary medical center, which may limit transferability to institutions with different organizational structures, staffing configurations, or resource conditions. Although core components of the intervention may be adaptable, contextual factors likely influenced both implementation pace and observed outcomes.

Second, the study employed a pragmatic quality improvement and implementation-oriented design without a comparator group or baseline-controlled evaluation. Accordingly, observed changes in care processes should be interpreted as indicators of implementation activity rather than evidence of causal effectiveness. The absence of controlled comparison limits attribution of observed improvements to the intervention alone.

Third, a central methodological limitation concerns the assessment of professional learning. Although CPD-oriented activities were intentionally embedded within the intervention, independent or prospectively defined educational assessments were not conducted. No validated competency instruments, pre–post knowledge measures, or structured evaluation of reflective practice were applied. Consequently, improvements in audit compliance and observed practice behaviors cannot be assumed to represent verified learning outcomes. The educational implications of the intervention therefore remain inferential.

Fourth, implementation outcomes were derived from routine audits, structured observational indicators, and documentation review. While iterative validation occurred during PDSA “Study” phases, these measures remain potentially subject to documentation bias and observer influence. In addition, qualitative observations were used as formative implementation reflections rather than subjected to formal qualitative analytic procedures, which constrains interpretive depth.

Fifth, patient-reported outcomes were exploratory in nature and based on a limited sample (*n* = 27) assessed at a single post-discharge time point. The absence of baseline measurement, comparator groups, and longitudinal follow-up restricts interpretation. Observed patterns in Emotional and Role Functioning domains should therefore be regarded as descriptive signals rather than evidence of intervention effect. Broader domains such as Social Functioning and Global Health Status may require longer follow-up and multi-context support to demonstrate meaningful change.

Finally, formal clinical quality endpoints—such as complication rates, readmissions, or cost-related indicators—were not systematically evaluated. The primary emphasis of the project was feasibility and implementation integration within routine practice. More robust evaluation of downstream clinical and educational outcomes would require larger samples, extended follow-up periods, and controlled or comparative designs.

Collectively, these limitations reflect inherent trade-offs in single-site, practice-embedded implementation research conducted in resource-constrained environments. Future multi-site and longitudinal studies incorporating independent educational assessment and comparative designs are warranted to more rigorously examine sustainability, scalability, and measurable clinical impact.

## 6 Conclusion

This study presents a practice-embedded, PDSA-guided Quality Stoma Practice Specific Program (QSPSP) as a pragmatic implementation-oriented approach within a resource-constrained clinical setting. The program integrated specialist nursing leadership, workplace-based learning activities, and iterative quality improvement cycles into routine care processes, demonstrating the feasibility of embedding CPD-oriented elements within everyday nursing workflows.

The findings suggest that PDSA-based implementation may provide a useful structural pathway for aligning professional learning, service delivery, and patient experience, although the study design does not permit causal attribution of observed improvements. Descriptive signals of change were observed in selected care process indicators and early post-discharge patient-reported outcomes; however, these findings should be interpreted primarily as implementation learning evidence rather than confirmation of CPD effectiveness.

More broadly, the work supports the conceptual shift toward viewing continuing professional development as a longitudinal, practice-integrated process embedded within clinical systems rather than as a standalone educational activity. The QSPSP illustrates a resource-sensitive model that may facilitate workforce capacity development and evidence translation in constrained healthcare environments, while allowing contextual adaptation.

Future research should further examine the sustainability, scalability, and cross-context applicability of practice-embedded CPD-informed implementation models using longitudinal, multi-site, and methodologically rigorous designs incorporating independent learning assessment and extended clinical outcome evaluation.

## Data Availability

The original contributions presented in the study are included in the article/supplementary material, further inquiries can be directed to the corresponding author/s.
